# Supplementary material for: The Effects of De Novo Mutation on Gene Expression and the Consequences for Fitness in Chlamydomonas reinhardtii
Source: Mol Biol Evol. 2024 Feb 16;41(3):msae035. doi: 10.1093/molbev/msae035 (PMC10910851; doi:10.1093/molbev/msae035)
Supplement: msae035_Supplementary_Data [file msae035_supplementary_data.pdf]

Supplementary materials

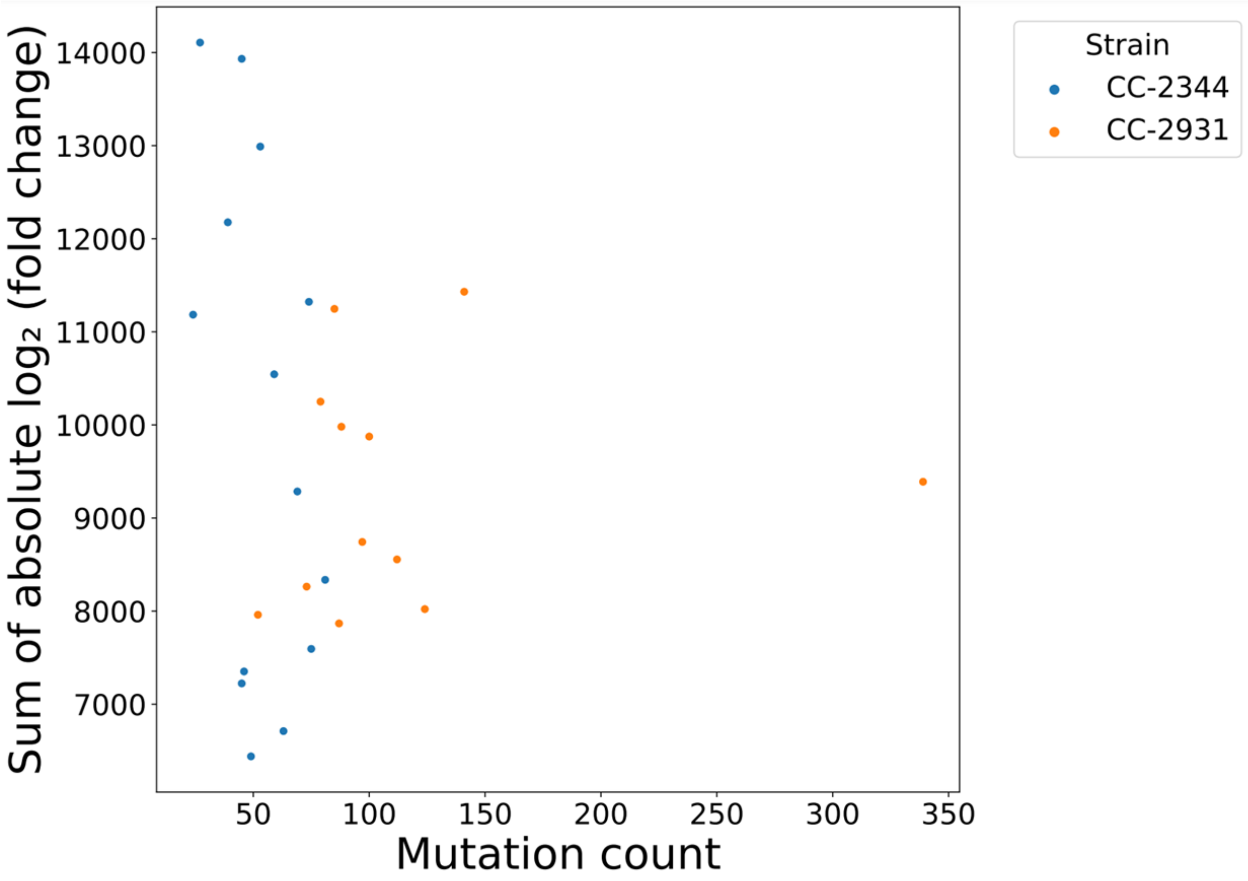

Fig S1: Scatterplot of the sum of the absolute log_2 fold change by mutation count. Expression change is measured as the sum of absolute log_2 fold change across all genes in an MA line and is plotted against the number of mutations in that MA line. There was no significant correlation found between the two variables (R^2 = 0.058, p = 0.421).

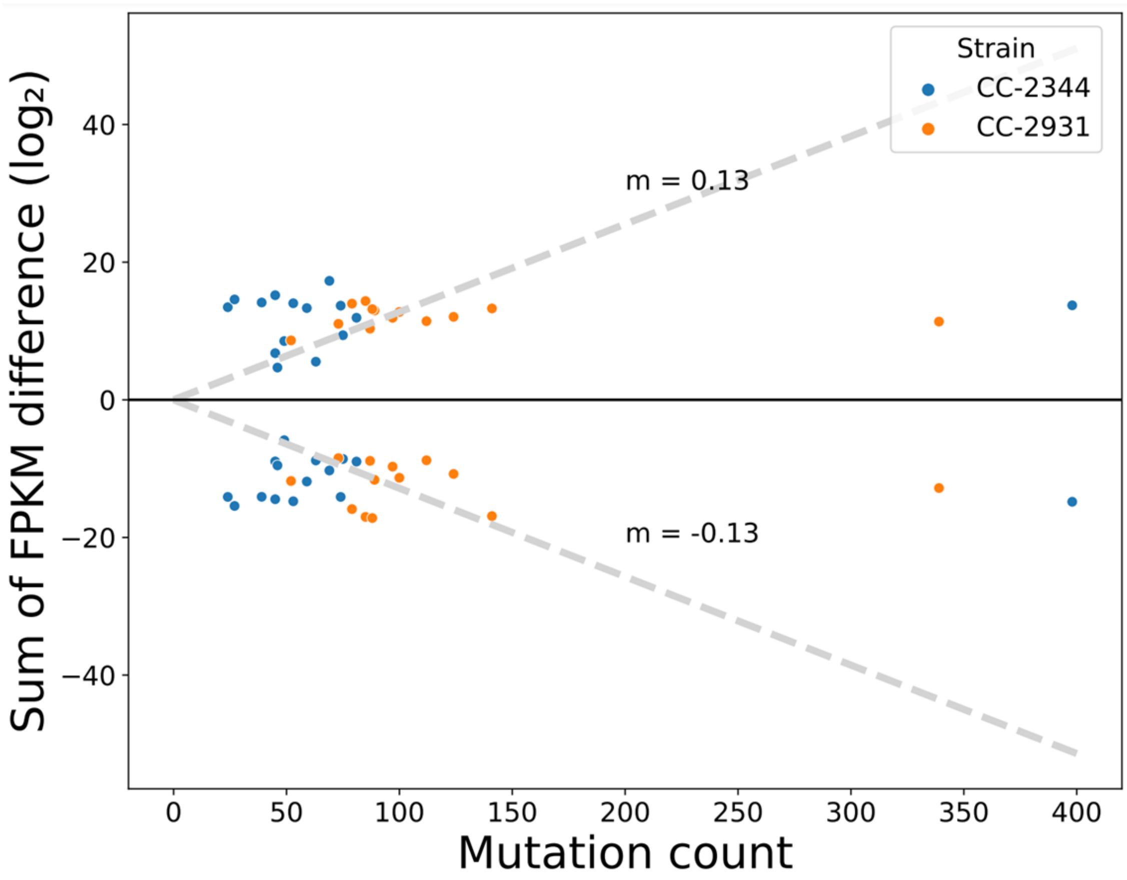

Fig S2. No correlation between the sum of FPKM differences and mutation count. Plot of the $\log_2$ transformed summed FPKM-normalized read count differences between MA lines and their respective unmutated ancestor. Only differentially expressed genes with $p_{adj} < 0.05$ were considered. Each MA line is shown twice with the same mutation count, points above the y=0 line represent upregulated genes and below the line are down-regulated genes. The dashed line represents the average rate at which expression changes per mutation. We did not find a correlation between the non-transformed differences in expression and mutation count (positive expression change ~ Pearson R = -0.0825, p = 0.676; negative expression change ~ Pearson R = -0.0607, p = 0.759).

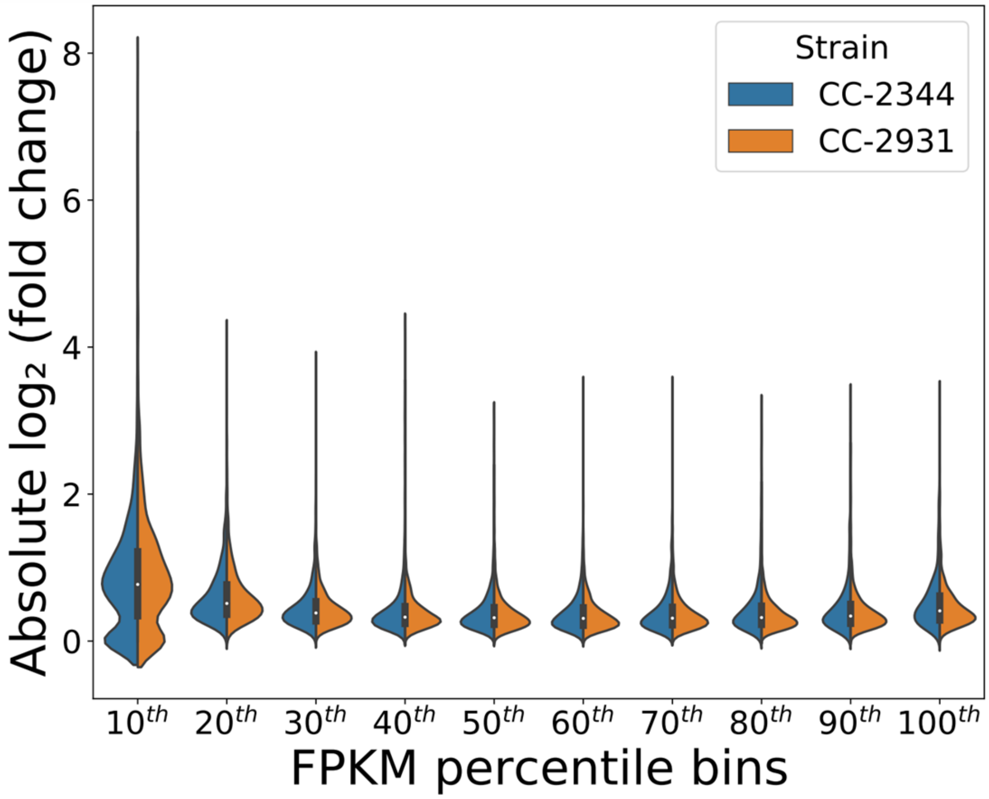

Fig S3. Ancestral expression level does not predict absolute expression change. Split-violin plot showing the distribution of genes in each FPKM-normalized ancestral expression bin ($n_{CC\text{-}2344}$ = 1745, $n_{CC\text{-}2931}$ = 1737). Each measurement within the distribution represents the median absolute expression change across all 28 MA lines per gene. There is no relationship between the ancestral expression level of a gene and its respective degree of expression change. The median expression change decreases from the 10th to 50/60th percentile expression bin and increases thereafter.

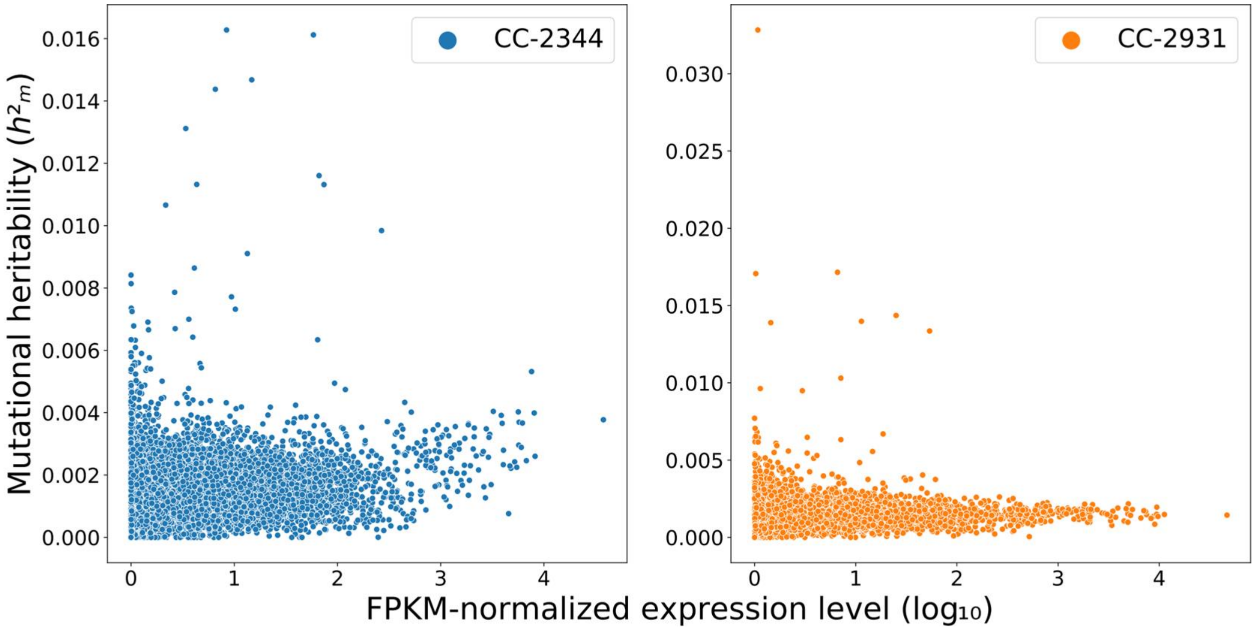

Fig S4. Slight positive correlation between mutational heritability and expression level. Scatterplot of the FPKM normalized gene expression counts of the ancestral line of each strain against its respective mutational heritability. The mutational heritability was estimated using the per generation mutational variance. There is a weak positive relationship for CC-2344 (Pearson's $R = 0.20$, $p = 1.22 \times 10^{-162}$) and CC-2931 (Pearson's $R = 0.06$, $p = 7.59 \times 10^{-14}$).

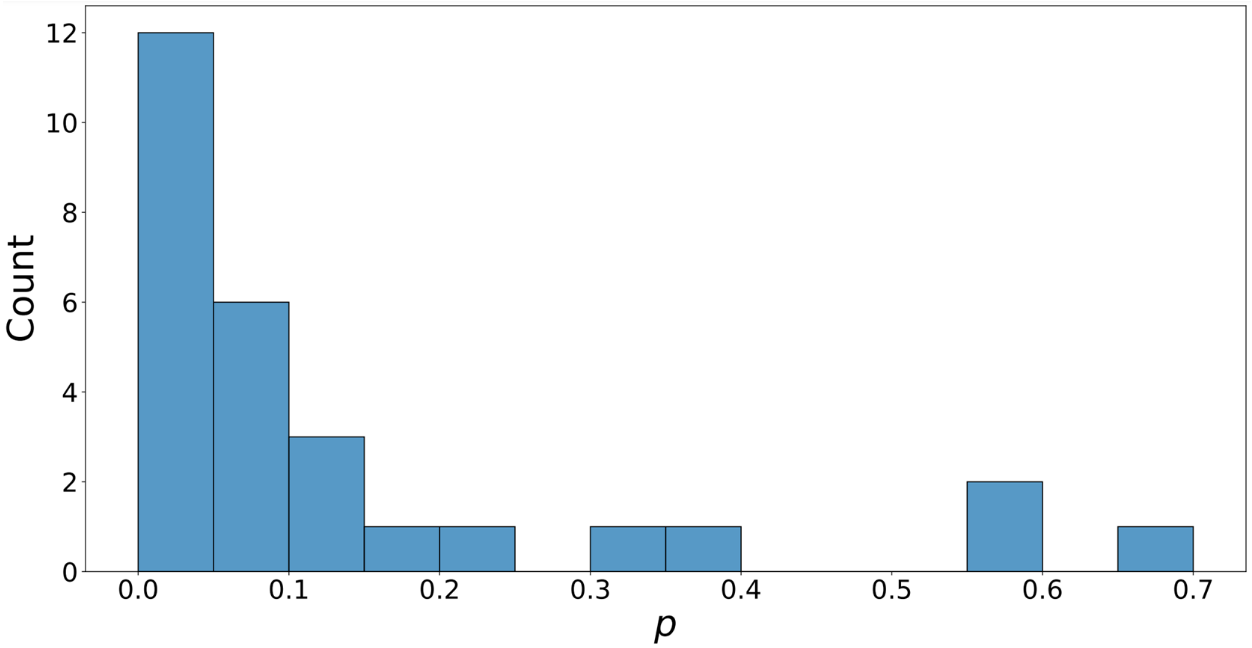

Fig S5. Overrepresentation of mutations within 100 bp of differentially expressed genes. Evidence of *cis*-regulatory mutations was inferred from an over-representation of mutations within 100 bp of

differentially expressed genes (DEGs). The observed distance between mutations and their nearest DEGs was compared to the expected distribution created by randomizing the DEGs across the genome over 10000 iterations. The *p* values represent the fraction of trials where the cases of simulated DEGs co-localizing with mutations was greater than those with observed DEGs. The distribution of *p* values is heavily right-skewed, showing the presence of an excess of mutations in or near DEGs in most MA lines.

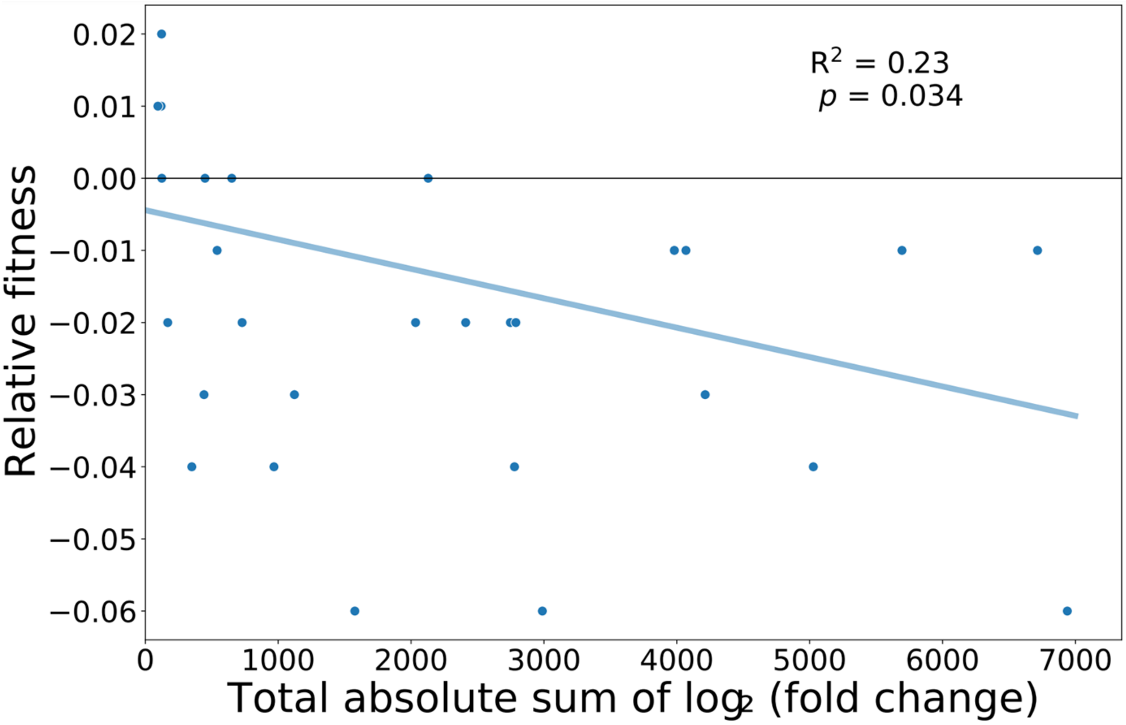

Fig S6. A negative correlation between relative fitness and total absolute sum of $\log_2$ fold change. Linear regression modelling the relationship between the total sum of absolute $\log_2$ fold change in differentially expressed genes and its fitness relative to the unmutated ancestor (R^2 = 0.23, *p* = 0.034).
